# Recent Progress on Near-Infrared Photoacoustic Imaging: Imaging Modality and Organic Semiconducting Agents

**DOI:** 10.3390/polym11101693

**Published:** 2019-10-16

**Authors:** Doyoung Jung, Suhyeon Park, Changho Lee, Hyungwoo Kim

**Affiliations:** 1 School of Polymer Science and Engineering & Alan G. MacDiarmid Energy Research Institute, Chonnam National University, 77 Yongbong-ro, Buk-gu, Gwangju 61186, Korea; 1993_jdy@naver.com; 2 Interdisciplinary Program of Molecular Medicine, Chonnam National University, 77 Yongbong-ro, Buk-gu, Gwangju 61186, Korea; suhyeonpark78@gmail.com; 3 Department of Nuclear Medicine, Chonnam National University Medical School & Hwasun Hospital, 264, Seoyang-ro, Hwasun-eup, Hwasun-gun, Jeollanam-do 58128, Korea

**Keywords:** photoacoustic imaging, near-infrared, contrast agents, organic semiconductors

## Abstract

Over the past few decades, the photoacoustic (PA) effect has been widely investigated, opening up diverse applications, such as photoacoustic spectroscopy, estimation of chemical energies, or point-of-care detection. Notably, photoacoustic imaging (PAI) has also been developed and has recently received considerable attention in bio-related or clinical imaging fields, as it now facilitates an imaging platform in the near-infrared (NIR) region by taking advantage of the significant advancement of exogenous imaging agents. The NIR PAI platform now paves the way for high-resolution, deep-tissue imaging, which is imperative for contemporary theragnosis, a combination of precise diagnosis and well-timed therapy. This review reports the recent progress on NIR PAI modality, as well as semiconducting contrast agents, and outlines the trend in current NIR imaging and provides further direction for the prospective development of PAI systems.

## 1. Introduction

Optical imaging modalities such as fluorescence imaging (FLI), multi-photon microscopy (MPM), optical coherence tomography (OCT), and diffuse optical imaging (DOI) are widely utilized in preclinical and clinical imaging field. These modalities have capabilities to provide real-time anatomical and functional images with superior resolution. In addition, by providing spectroscopic information, it is possible to obtain information of the constituent materials in/ex vivo. Additionally, they are nonionizing imaging methods and relatively cost-effective to fabricate and maintain its performance [1,2,3,4]. Unfortunately, the penetrating depth of optical imaging cannot reach to over ~1 mm in biological tissue because of the scattering and absorption of light in tissue. In general, microscopic techniques show only by ~500 µm [5]. In particular, OCT based on interferometric devices enables to image a little bit deeper depth by ~2 mm in skin, retina, and cornea regions [6,7]. DOI overcomes this limitation of imaging depth using the diffused light property and achieves the several centimeters of imaging depth in breast and brain regions. However, DOI should scarify their spatial resolution because they have to experience the multiple light scattering and absorption in tissue medium [8,9]. Thus, because pure optical imaging techniques has a tradeoff between the penetrating depth and the spatial resolution, there is a need to developing new imaging techniques by fusing different characterized imaging modalities. 

**Figure 1:**
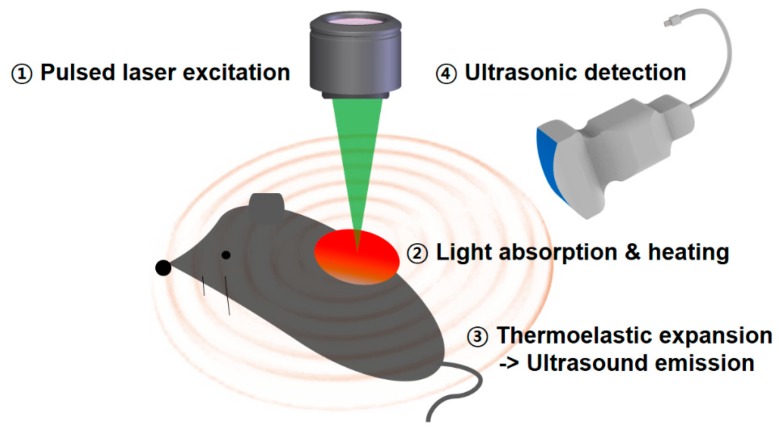
Schematic description of the generation of a photoacoustic signal.

Photoacoustic imaging (PAI) is currently considered a promising hybrid imaging modality that features integrated-imaging properties for both optical and ultrasound imaging techniques; it is already utilized in a diverse range of preclinical and clinical fields. Based on dual-imaging characteristics, PAI is capable of representing deep regions while maintaining high ultrasonic resolution. Figure 1 describes the principle of PAI. When a nanosecond-pulsed laser is illuminated into a sample with absorbing chromophores, they absorb the light energy and generate heat. The increase in temperature due to heat causes thermal elastic expansion, thereby leading to the generation of acoustic waves in the tissue; this effect is known as the photoacoustic (PA) effect. By sensing these propagating acoustic waves with conventional ultrasonic transducers, PAI allows the mapping of the location of the absorber in the biological tissue [10,11,12,13,14,15,16,17]. Because the scattering and the speed of ultrasound are less than those of light, PAI allows deep-tissue imaging.

The ability of PAI to discern the morphological (i.e., vasculature networks, distributions of fat and melanin, tendon conditions, cellular structure, etc.) and physiological factors (i.e., concentrations of hemoglobin, saturated oxygen levels, blood velocity, metabolism ratios, etc.) of biological tissues is excellent while choosing the optimal laser wavelength of natural chromophores such as oxy- and deoxy-hemoglobin, fat, collagen, protein, melanin, and water [10,11,18,19,20,21,22]. In addition, by injecting exogenous contrast agents into the body, colorless main organs, such as the sentinel lymph nodes (SLNs), guts, and bladder, which have relatively poor absorption coefficients, can be targeted and visualized based on the molecular PAI approach [23,24,25,26,27]. Furthermore, by cooperating with multifunctional agents for therapy and diagnosis, PAI can contribute to precision medicine [28,29,30,31]. Owing to these advantages, PAI can play a vital role in advancing fundamental research and solving real clinical issues [32,33,34,35].

Even if the natural absorbing biomolecules of the biological tissue offer a diverse contrast to PAI, the absorption peaks normally position in the visible spectrum (i.e., 400–650 nm). Visible light is incapable of penetrating into the deep-lying areas because it undergoes a high level of scattering and absorption [36,37]. In short, the natural absorbing biomolecules for the multiple contrast of PAI only allow for the visualization of areas at a limited depth. To resolve this issue, near-infrared (NIR) light can be used as the PAI laser source, and exogenous contrast agents with strong absorption spectra in the NIR region are proposed as solutions to achieve deep-tissue imaging [38,39].

In regard to the exogenous contrast agents, many contrast agents have been developed thus far for PAI in biomedical or clinical applications; however, only those demonstrating a strong absorption in the NIR region can be used in imaging in the NIR window. Recently, as the significance of NIR PAI has attracted ever-increasing attention, semiconducting polymers that show a narrow band gap resulted from extended π-conjugation have been extensively researched due to their broadband absorption as a main platform for imaging contrast agents. During preparation, the polymers are normally encapsulated within biocompatible polymer shells, which forms core–shell-type, semiconducting polymer nanoparticles (SPNs) that are stable under aqueous conditions in addition to being not cytotoxic. 

Therefore, in this review, we introduce the recent progress on the development of PAI modality and contrast polymeric agents for the NIR imaging, which can help readers to grasp the recent trend and will be a guideline to the future development of new imaging applications.

## 2. Interaction of Near-Infrared Light with the Biological Tissue

**Figure 2:**
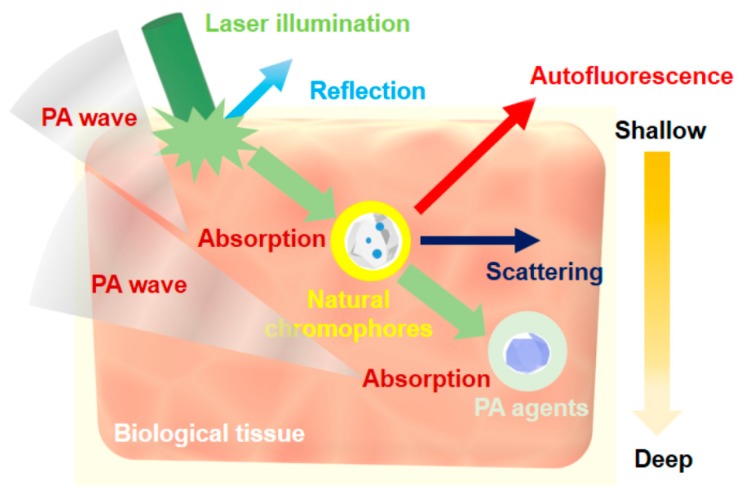
Conceptual depiction of general interactions between light and tissue.

When light propagates into biological tissue, several events occur between light and tissue such as reflection, absorption, auto-florescence, and scattering [40], as shown in Figure 2. In particular, scattering and absorption are a critical factor to determine the imaging depth of PAI. Typically, if an illumined laser beam can penetrate without any energy loss due to scattering or absorption, then deep PAI imaging can be obtained easily. However, the absorption coefficients of whole blood are predominantly positioned between 200 and 600 nm, whereas lipids have some peaks near 980 nm. These absorption peaks are beneficial for specific imaging of blood vessels and plaques but hinder deep-tissue imaging. However, these absorption coefficients rapidly decrease and mostly disappear over the NIR region (i.e., 700–1600 nm). Many PAI studies based on deep-tissue imaging have been conducted using contrast agents such as organic materials (e.g., ICG or methylene blue) and inorganic materials (e.g., carbon-based nanoparticles) in the NIR-I window (i.e., 700–1000 nm) due to the relatively low absorption by whole blood [24,28,41,42,43]. However, the NIR-I window is not an optimal spectrum for deep-tissue PAI because of its scattering factor [40]. Although most human tissues have scattering coefficients that exponentially decrease beyond 700 nm, they still show a low level of scattering beyond 1000 nm. Additionally, when considering the NIR-II window (i.e., 1000–1600 nm), water has significant absorption above 1400 nm; the absorption coefficient of water increases continuously from 500 nm and becomes larger than that of biological tissues above 1200 nm [44]. Thus, considering biological absorption or scattering as well as water absorption, it is ideal to position the deep-imaging window in the range of 1000–1200 nm.

## 3. Photoacoustic Imaging Systems with Near-Infrared Light

Depending on the field of application, various types of PAI systems can be applied. In terms of spatial resolution and imaging depth, they can be classified as either photoacoustic microscopy (PAM) [45] or photoacoustic tomography (PAT) [46]. Typically, PAM can delineate micro-sized samples such as cells and blood microvessels with various micro-spatial resolutions and high sensitivity. According to the strategy of achieving high spatial resolution, PAM is also divided into (i) optical-resolution PAM (OR-PAM) that provides a high spatial resolution using optical techniques such as a tiny focused beam [47] and (ii) acoustic-resolution PAM (AR-PAM) that realizes high ultrasonic resolution by using a focused ultrasound-capturing configuration [48]. Although the imaging depth demonstrated by PAM with the aid of low-scattering ultrasound detection is better than that demonstrated by conventional microscopic imaging modalities, PAM only visualizes the regions at relatively shallow depths. Therefore, it is not appropriate for deep-tissue, clinical implementation. PAT can reveal deeper regions owing to its systemic advantages with a low-frequency transducer, reconstruction algorithms, and a clinically used ultrasound imaging (USI) system [49]. Although it cannot discern micro-sized objects, it is an emerging imaging tool in the clinical field. PAT is divided into (i) photoacoustic computer tomography (PACT) [50] and (ii) clinical USI/PAI [17] based on the system specifications. In general, PACT utilizes a multi-array transducer in the form of a ring, a sphere, or an arch to quickly acquire multi-directional acoustic signals and uses reconstruction algorithms to generate the volumetric image. Clinical USI/PAI can systemically utilize the conventional USI system. By attaching a laser-beam-delivery, fiber-optic bundle to a USI probe, structural USI and functional PA images can be simultaneously acquired and facilely applied to clinical applications. In Section 3.1, we introduce the representative development of PAM systems with NIR light. Using NIR light, PAM imaging of relatively deep regions was achieved with a high spatial resolution. In Section 3.2, three different PAT systems using NIR light are summarized with system schematics and representative images. Table 1 shows representative PAI systems with NIR light. PAI systems are classified on the basis of system type, wavelength used, imaging depth, spatial resolution, detector type, and application.

### 3.1. High-Resolution Photoacoustic Microscopy with Near-Infrared Light

**Figure 3:**
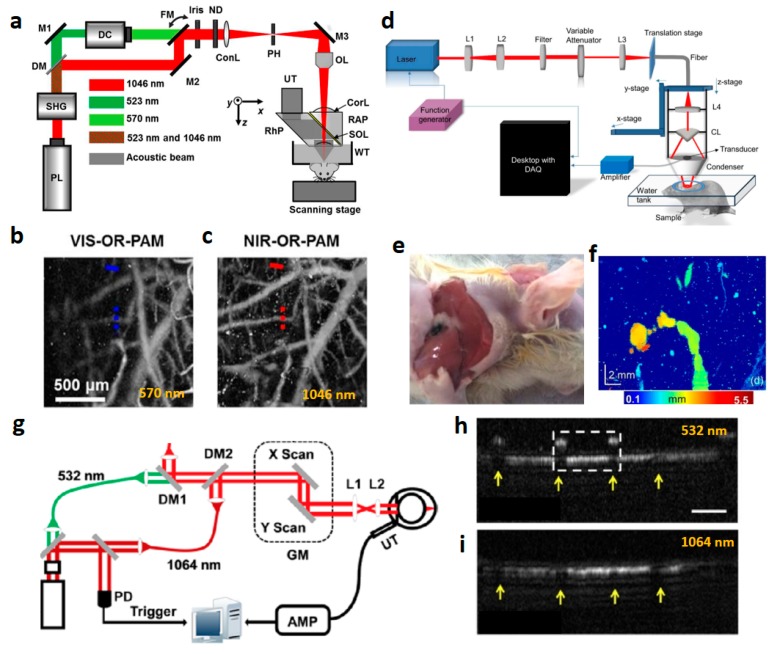
Photoacoustic microscopy (PAM) with NIR light. (**a**) Schematic of NIR optical-resolution photoacoustic microscopy (NIR-OR-PAM). (**b**,**c**) OR-PAM images of a mouse brain of VIS-OR-PAM and NIR-OR-PAM, respectively. (**d**) Schematic of NIR acoustic-resolution photoacoustic microscopy (NIR-AR-PAM). (**e**,**f**) Photography and AR-PAM image of sentinel lymph nodes (SLNs) with a black ink, respectively. (**g**) Schematic of NIR optical resolution-photoacoustic ophthalmoscopy (OR-PAO). (**h**,**i**) NIR-OR-PAO images of a mouse retinal blood vessels at 532 nm and 1064 nm, respectively. Reprinted with permission from [52,53,56]. Copyright, The Optical Society America (2014) [53] and (2012) [52], and John Willey and Sons (2019) [56].

Figure 3a depicts the schematic of NIR OR-PAM [63]. The performance of OR-PAM is predominantly dependent on the specifications of the optical setup, such as an objective lens. In particular, by removing the spatial noise of the laser beam using a pinhole (PH), a high-quality beam is generated. After passing through an objective lens (OL), the beam is focused onto the sample. Through the generation of PA signals from a tiny focused beam area, a micro-scale resolution can be achieved. Also, the focused ultrasonic detection part contributes the high sensitivity. To compare the imaging depth performance between visible light and NIR light, 570 nm and 1046 nm laser systems were set up and tested by imaging the same mouse brain area. Under NIR light excitation, the maximum imaging depth increased by 3.2 mm while maintaining a 6 dB SNR. Additionally, OR-PAM at 1046 nm (Figure 3c) showed more clear brain blood vessels than OR-PAM at 570 nm (Figure 3b). Unlike OR-PAM, AR-PAM generates a high resolution using focused ultrasound detection. Figure 3d depicts the schematic of NIR-AR-PAM. A 1064-nm laser was focused onto a conical lens (CL) and refocused by a condenser [56]. The focused transducer was installed in the condenser, so that it can directly capture the PA signals with high spatial resolution. By using a black tape in chicken breast tissue, almost 11 mm imaging depth was demonstrated. Based on the same NIR-AR-PAM configuration, an invisible sentinel lymph nodes (SLNs) was visualized with a black ink injection, as shown in Figure 3e,f. Figure 3g presents the system setup of NIR light optical-resolution photoacoustic ophthalmoscopy (OR-PAO) that utilizes a focused laser beam and unfocused ultrasound detection. Thus, even though PAO is considered a form of OR-PAM, it has the drawback of lower sensitivity caused by the unfocused ultrasonic transducer (UT) [52]. To achieve dual-wavelength beam scanning, a dichroic mirror (DM1 ,2) integrated the 532-nm visible and 1064-nm NIR laser. Fast volumetric scanning with two-dimensional optical scanners (GM) was carried out with the collimated beam to visualize the mouse retinal area. Finally, the unfocused needle-type UT (central frequency 35 MHz) detected PA signals. Figure 3h,i show the 532-nm and 1064-nm OR-PAO images, respectively. Due to strong absorption of hemoglobin at 532 nm, shadows of blood vessels on the retinal layers in the white dashed box disturbed the visualization of inner retinal blood vessels. Fortunately, because of the lower absorption and scattering of the 1064-nm laser beam in hemoglobin, the NIR light OR-PAO is sufficient to show inner blood vessels clearly. Therefore, utilization of NIR light in PAM imaging contributes to improving the depth and resolution of PAM.

### 3.2. Deep-Tissue Photoacoustic Tomography with Near-Infrared Light

**Figure 4:**
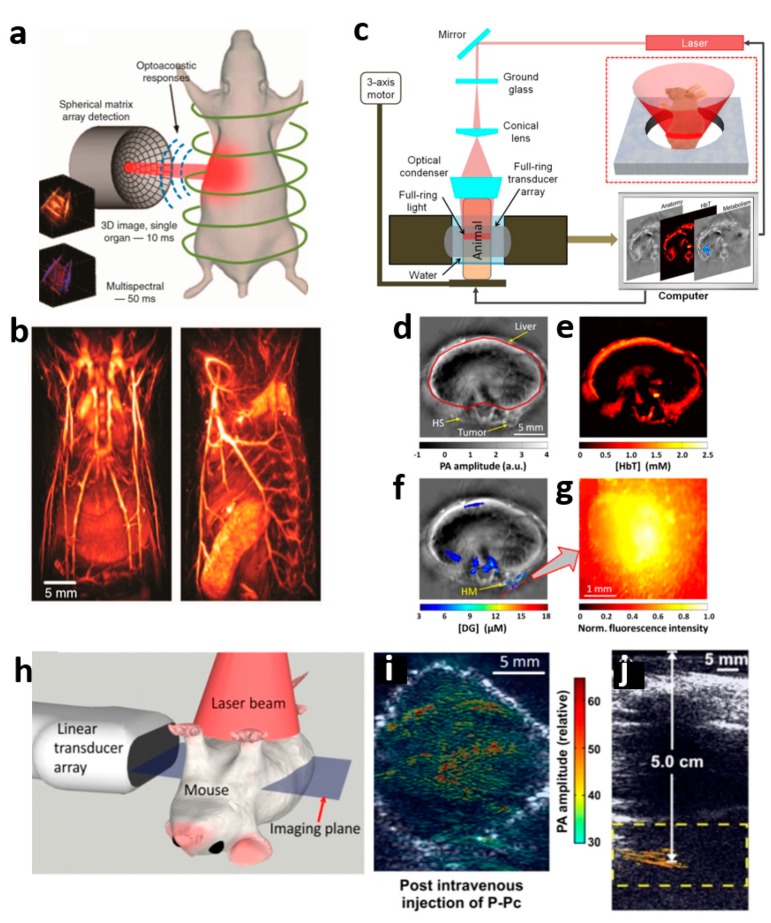
Deep-tissue PAT systems. (**a**) Schematic of spiral volumetric photoacoustic computed tomography (SV-PACT). (**b**) 3D whole body mouse SV-PACT image with high spatial resolution. (**c**) Schematic of ring-shaped confocal PACT (RC-PACT). (**d**) Anatomical RC-PACT image of mouse at 776 nm. (**e**) RC-PACT image of hemoglobin (HbT) concentration. (**f**) PACT image and (**g**) Florescence image of IRDye800-2DG concertation in tumor. (**h**–**j**) Deep PAI and USI images of the tumor and human arm with phosphorous phthalocyanine (P-Pc) formulation, respectively. Reprinted with permission from [57,58,62]. Copyright, SPIE (2012) [58], Ivyspring (2016) [62], and Nature Publishing Groups (2017) [57].

Figure 4 illustrates spiral volumetric photoacoustic computed tomography (SV-PACT or SV-OT) for visualizing volumetric dynamics in mice in real time [57]. Figure 4a depicts the diagram of the spiral volumetric PACT system. To generate PA signals, the optical parametric oscillator (OPO) with a 10 ns pulsed width, 30 mJ energy, and 100 Hz repetition rate was used. To image the dynamics of blood vessels, the selected laser wavelengths of 730, 760, 800, 850, and 900 nm were excited on the target. A spherical matrix transducer composed of 256 elements (4 MHz, 40 mm radios) was utilized to capture the PA signals at multiple locations. Owing to its three systemic advantages such as the NIR laser source, the multi-arrayed ring transducer, and fast spiral trajectory scanning, SV-PACT can achieve whole-body small-animal PA images without any invisible regions at 100 volumes per second. Finally, using a universal back-projection algorithm, a whole-body mouse SV-PACT image was acquired, as depicted in Figure 4b. Figure 4c indicates the schematic of a ring-shaped confocal PACT (RC-PACT) system. This system was tested to acquire volumetric PA images of a mouse tumor glucose metabolism [58,59]. A tunable laser based on Ti-sapphire from 680 to 990 nm was also used in this system to achieve deeper penetration. Subsequently, it was diffused by ground glass (EDC5, RPC Photonics) and a donut-shaped beam was generated by a conical lens. This system utilized reliable energy (below 15 mJ/cm^2^) and a relatively low-frequency full ring-shaped transducer (5 MHz) for whole-body mouse imaging. The ring-shaped transducer array composed of 512 elements had a 50 mm ring radius. Each element was designed to generate 19 mm axial focal depth. As depicted in Figure 4d–g, RC-PACT was used to evaluate the glucose metabolism of the tumor. First, the anatomical image was acquired using a 776-nm laser, which showed tumors, a healthy kidney, and a liver (Figure 4d). Second, by applying three wavelengths (i.e., 776, 796, and 820 nm), hemoglobin (HbT) concentration was acquired (Figure 4e). Finally, by injecting IRDye800-2DG, the tumor glucose metabolism was successfully mapped (Figure 4f), and in addition, a tumor with IRDye800-2DG was observed with fluorescence imaging as shown in Figure 4g. Figure 4h depicts the deep PA imaging application using the clinical PAI/USI system at 1064 nm [62]. This approach was developed based on the clinical USI system. By combining the optical fiber bundle for 1064-nm laser delivery and the USI imaging probe, the PAI and USI images can be visualized simultaneously. Therefore, this method is more powerful for application in the real clinical field and has already been utilized in several clinical diagnostic applications, such as for thyroid cancer, sentinel lymph node detection, breast cancer, and diabetic foot [63]. To improve the deep penetrating capability, phosphorus phthalocyanine (P-Pc) formulation, which has a high absorption peak at 1064 nm, was used in the tumor (Figure 4i) and the human arm (Figure 4j). As shown in Figure 4i, P-Pc formulation shows an excellent PA signal at the inner tumor area. The 1064-nm PAI/USI system detected deep PAI images with the tube containing P-Pc formulation from the opposite site of the human arm. As depicted in Figure 4j, this system was able to detect the tube up to 5.0 cm.

## 4. Organic Semiconducting Materials for Near-Infrared Imaging

### 4.1. General Design Strategy for the Contrast Agent

**Figure 5:**
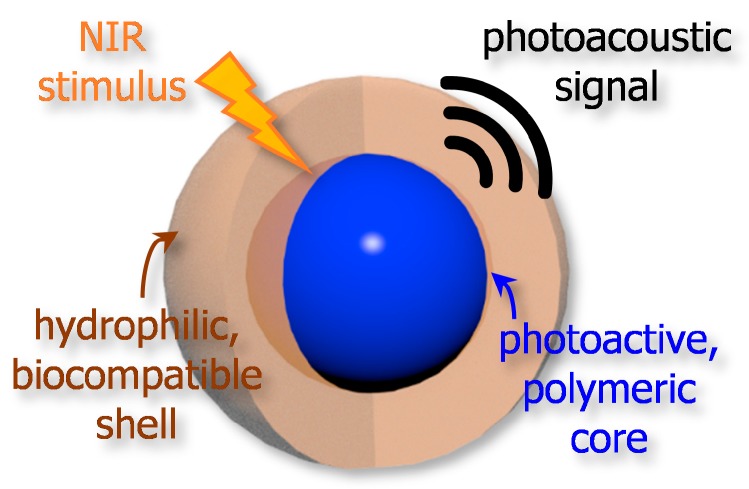
Illustration of general design concept for a core–shell-type, polymeric contrast agent for NIR photoacoustic imaging.

Most contrast agents have been designed to form a core–shell-type structure where polymers play crucial roles, as depicted in Figure 5. In general, the core part consists of organic semiconducting materials that generate a photoacoustic signal in response to an NIR light while polymers at the shell are required to be hydrophilic and biocompatible. The size of resulting core–shell particle typically ranges from nanometers to a few microns. Thus, the particles can be appropriately applied in bio-imaging, resulting in a stark contrast in the photoacoustic signal as an imaging agent when irradiated by NIR light [44,64,65,66].

We discuss and summarize the core materials in the sections below. In brief, the materials mainly include abundant π-conjugated polymers together with small molecules or other inorganic materials such as carbon materials or metal complexes, which are further classified by an NIR light source that they absorb for the generation of photoacoustic signals. Majority of the imaging agents that have been extensively studied thus far only absorb the light in the NIR-I region (wavelength, 700–1000 nm). However, to increase the penetration depth and reduce the background signals, many recent studies have focused on the use of light sources in the NIR-II region (wavelength, 1000–1600 nm), demonstrating enhanced imaging performance—for example, deep-tissue imaging or high-resolution imaging [67,68]. 

**Figure 6:**
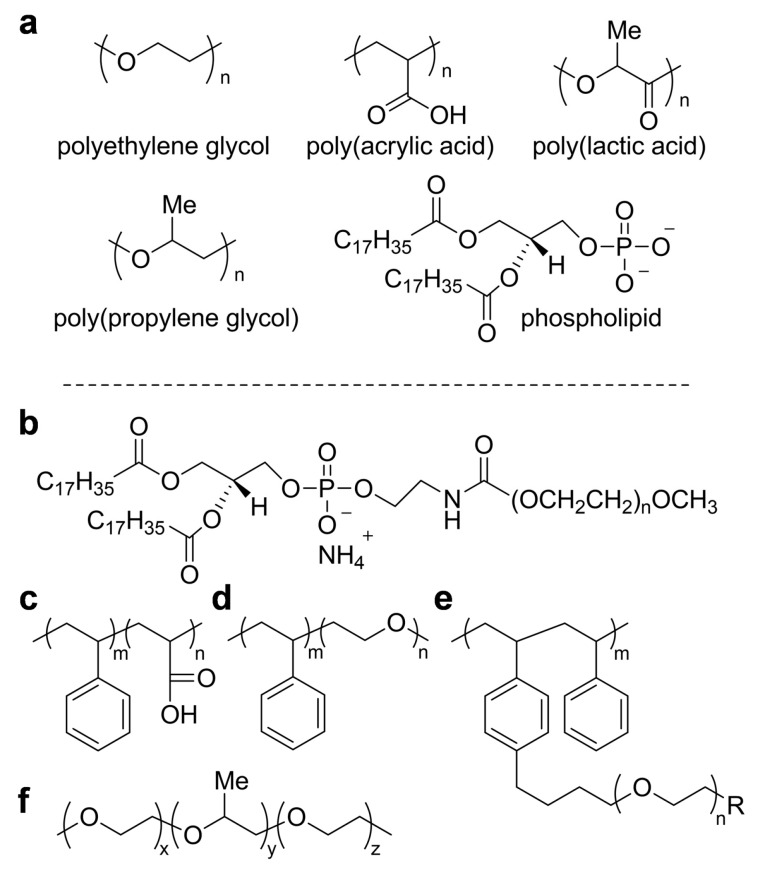
(**a**) Typical basic polymer components that cause hydrophilicity in the materials, and (**b**–**f**) select block copolymers from the basic components which have been notably used.

For the shell materials, diverse biocompatible polymers can be used including conventional hydrophilic components, such as polyethylene glycol (PEG), poly(acrylic acid) (PAA), poly(lactic acid) (PLA), polypropylene glycol (PPG), and phospholipids, as illustrated in Figure 6a, which can result in various further combinations, leading to copolymer structures—for example, block copolymers or branched polymers, as depicted in Figure 6b–f. Recently, the polymeric agents have further advanced to demonstrate not only the in situ optical detection capability, but also therapeutic functions, leading to multi-functional agents that give rise to theragnosis—an emerging combined concept of simultaneous diagnosis and therapeutics [23,69,70,71,72,73].

### 4.2. NIR-I Imaging Contrast Agents

#### 4.2.1. Semiconducting Polymers

Semiconducting polymers have been extensively used as a photoactive core material, because their optoelectric properties as well as surface properties can be widely tailored for desired applications. In particular, modification of chemical structures in the polymer backbones causes significant change in the band gap of polymers, which results in narrow-band-gap polymers that absorb light in the NIR region. As the effective conjugation length increases, the absorbance in the NIR region is intensified. Furthermore, biocompatible polymer components can be used to encapsulate the core polymers, or they can be directly tethered onto the backbone of polymers as a pendant group, which reduces cytotoxicity and improves solubility or dispersibility under biological conditions.

**Figure 7:**
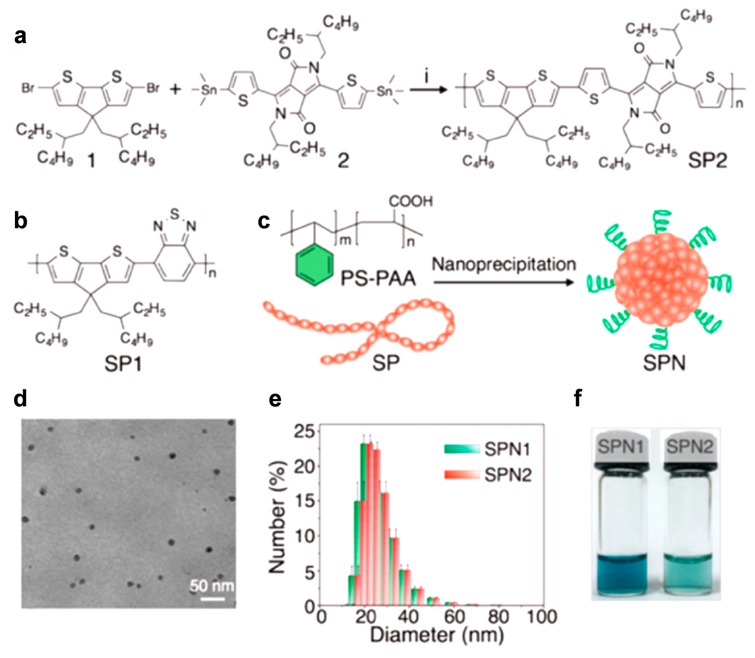
Synthesis and characterization of semiconducting polymer nanoparticles (SPNs). (**a**) Synthetic route of SP2 via Stille polymerization under the reaction conditions (*i*) PdCl_2_(PPh_3_)_2_ and 2,6-di-*tert*-butylphenol, 100 °C for 12 h. (**b**) Chemical structures of SP1. (**c**) Schematic illustration of synthesis of SPNs. (**d**) Representative TEM image of SPNs: SPN2. (**e**) Representative dynamic light scattering (DLS) profiles of SPNs. (**f**) Photos of SPN solutions (18 μg·mL^−1^). Reprinted with permission from [74]. Copyright, American Chemical Society (2016).

Pu et al. notably demonstrated semiconducting contrast agents for NIR-I imaging, considering the fundamental concept of π-conjugated system (Figure 7a,b) [74]. The designed agent particles comprise core semiconducting polymers (SP1 and SP2), and block copolymer shells via nanoprecipitation (Figure 7c). The spherical particles exhibited a uniform morphology, and the diameters of the agents measured an average of 25 nm (Figure 7d,e). The resulting agents absorbed NIR light at 780 nm and exhibited good water dispersibility (Figure 7f). Furthermore, the authors used the agent not only for optical imaging but also for photothermal therapy. After introducing a targeting moiety (anti-TRPV1, TRPV1: transient receptor potential cation channel subfamily V member 1) on the surface of the particles through amide bond formation, they were able to demonstrate the spatiotemporal, selective control of Ca^2+^ flux in a cation channel of TRPV1 as converting light energy into heat on the local designated area of TRPV1.

**Figure 8:**
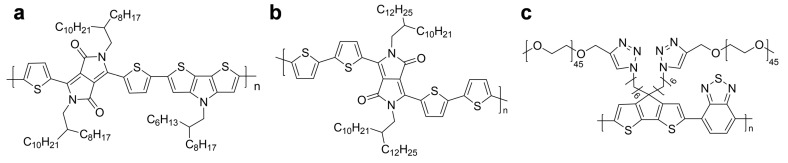
Chemical structures of semiconducting polymers that simultaneously generate a photoacoustic signal and heat for photothermal therapy in response to NIR-I light.

Other semiconducting polymers that are characterized by dual photophysical properties have also been reported. Liu et al. demonstrated agent nanoparticles based on a conductive polymer, as depicted in Figure 8a. In the polymer backbone, strong intermolecular charge transfer between an electron-rich donor and an electron-deficient acceptor occurred, which significantly red-shifted the absorption spectrum of the entire backbone. Therefore, when excited at 808 nm, this nanoplatform facilitated photoacoustic imaging (PA) and photothermal therapy (PTT) for cancer theragnostics in a manner superior to that by conventional PA/PTT agents, such as ICG (indocyanine green) [75]. 

Size of the photoactive core affects the absorbance of contrast agents. Wu et al. found that the core size of a particle that was comprised of a semiconducting polymer (Figure 8b) notably altered its absorption spectrum as well as its molecular weight, because of bending or kinking of the π-conjugated backbone that adjusted the effective conjugation length [76]. In general, an increase in the core size or molecular weight of the polymer was found to red-shift the absorption spectrum of the whole particle. Thus, they could fine-tune the absorption maxima of the core dots from 630 to 811 nm through facile manipulation. Furthermore, after encapsulating the core with a PEG-based amphiphilic polymer, they could use the resulting material in PA/PTT application for in vivo cancer treatment.

Figure 8c depicts a semiconducting polymer grafted with PEG chains, which results in an amphiphilic copolymer and formed single-component nanoparticles via a self-assembly process under physiological conditions without the need for an auxiliary polymer component [77]. The backbone of the polymer has π-conjugated system and shows hydrophobicity as well. Thus, it can absorb NIR light and produce a photoacoustic signal (PA) as well as heat (PTT) as expected; hydrophobic drugs (doxorubicin) can be loaded in the core owing to the hydrophobic interaction and π–π interaction, which enable the in situ chemotherapy of cancer in living mice.

**Figure 9:**
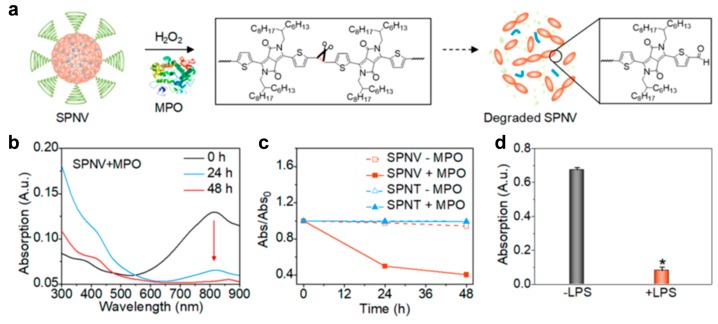
In vitro biodegradability study of semiconducting polymer nanoparticles (SPNs). (**a**) Schematic illustration of the degradation of SPNV (SPN with vinylene groups) in the presence of myeloperoxidase (MPO) and H_2_O_2_. (**b**) Absorption spectra of SPNV in the presence of H_2_O_2_ (300 μM) and MPO (50 μg mL^−1^) at 37 °C for 0, 24, and 48 h in phosphate buffer (50 mM, pH = 7.0) containing NaCl (150 mM). (**c**) Absorption decrease (Abs/Abs_0_) of SPNV at 819 nm and SPNT (SPN without vinylene groups) at 828 nm in the absence or presence of MPO (50 μg mL^−1^) and H_2_O_2_ (300 μM) as a function of incubation time. [SPNs] = 3 μg mL^−1^. (**d**) Absorption intensity of SPNV at 819 nm after incubation with RAW264.7 cells (276,000 cells mL^−1^) treated with or without lipopolysaccharides (LPS). Error bars were based on the standard deviations (SD) of three parallel samples. * Statistically significant difference in cells treated with and without LPS (p < 0.005, n = 3). Reprinted with permission from [78]. Copyright, American Chemical Society (2018).

Removal of exogenous agents is mandatory after the end of life. Hence, the contrast agents need to have relevant retention time without bioaccumulation or degradation in a biological system. Figure 9 illustrates a demonstration of a biodegradable contrast agent (SPNV). Many degradable units, such as esters or amides, prevent the overlap of p orbitals and frustrate the delocalization of electrons. However, the incorporation of vinylene units in the backbone of polymer prolonged the electronic conjugation and even enhanced the absorption coefficient. Furthermore, the functional group degraded into monomeric aldehyde compounds in response to hypochlorous acid (HClO), a strong oxidant generated by myeloperoxidase (MPO) and hydrogen peroxide (Figure 9a,b). The agent without vinylene units (SPNT) was non-responsive and stable under the oxidation conditions (Figure 9c). Biodegradability of SPNVs was further demonstrated in macrophage cells (RAW264.7) that can activate MPO when triggered by lipopolysaccharides (LPS). The considerable amounts of SPNV were removed as designed (Figure 9d) [78].

**Figure 10:**
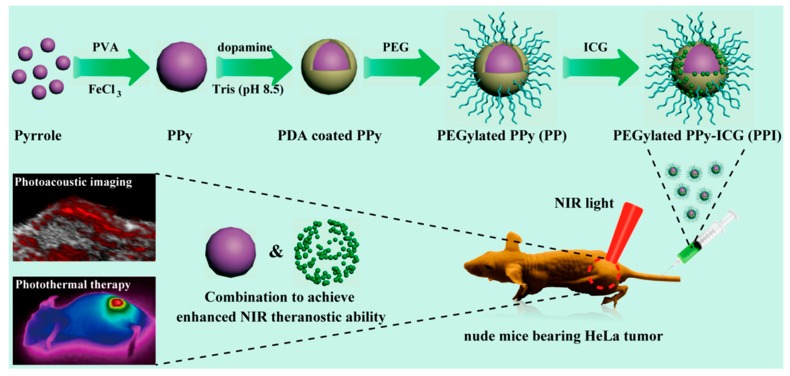
Schematic illustration of the formation and NIR theragnostic applications of PEGylated indocyanine green (ICG)-loaded polypyrrole nanoparticles (PPI NPs). Reprinted with permission from [81]. Copyright, American Chemical Society (2019).

For facile preparation, polypyrrole can be used to design the photoacoustic contrast agent [79,80]. Recently, Liu et al. developed agent capsules using polypyrrole particles that were coated with polydopamine (PDA) and PEG for biocompatibility. Furthermore, the agent was loaded with indocyanine green (ICG), which increased the efficiency of the material. Therefore, PEGylated, ICG-loaded polypyrrole nanoparticles (PPI NPs) demonstrated enhanced photoacoustic and photothermal abilities (Figure 10) [81].

#### 4.2.2. Semiconducting Small Molecules

**Figure 11:**
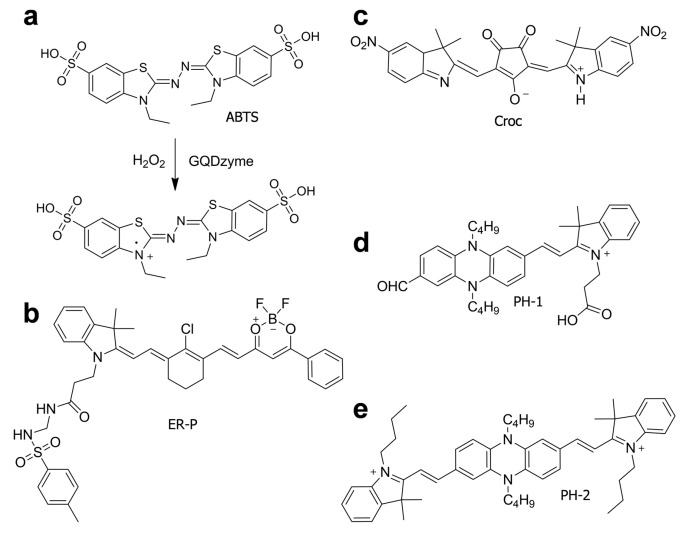
Chemical structures of small-molecule agents for NIR-I photoacoustic imaging.

Semiconducting small molecules have also been used for the fabrication of NIR-I contrast agents. In general, they are chemically defined and have shorter conjugation lengths than those in semiconducting polymers. However, they demonstrate a strong push–pull effect, which promotes the overlap of p orbitals and causes effective conjugation. Figure 11 illustrates example chemical structures of the small molecules. Nie et al. reported that 2,2′-azino-bis (3-ethylbenzothiazoline-6-sulfonic acid) (ABTS) exhibited strong absorbance in the NIR region when oxidized (Figure 11a) [82]. Thus, they prepared an exosome-like vesicle that contains ABTS and graphene quantum dot nanozyme (GQDzyme) and exhibits a peroxidase-like activity. In the presence of hydrogen peroxide, the GQDzyme converted ABTS to the oxidized form, activating the photoacoustic ability. The H_2_O_2_-sensitive agent was further functionalized with folic acid (FA) and natural erythrocyte membranes (RM) to mimic biological exosomes. Therefore, the vesicle agent demonstrated biocompatibility and stealth ability during long-term circulation, and enabled deep-tissue imaging in response to H_2_O_2_ produced from nasopharyngeal carcinoma (NPC) cells.

Figure 11b illustrates a dual-mode probe that emits not only fluorescence but also a photoacoustic signal [83]. Furthermore, the probe (EP-R) was found to have two absorption peaks at 700 and 800 nm, and the resulting photophysical properties of the probe were strongly dependent on the polarity of the medium. Therefore, authors could use the probe for ratiometric sensing of diabetes-induced liver injury, in which the ratio between hydrophobic and hydrophilic domains in the endoplasmic reticulum (ER) changes and cellular polarity increases.

Small molecules can form nanoparticles through a self-assembly process. For example, a croconine (Croc) dye formed a self-assembled complex with human serum albumin (HSA) without the need for exogenous biocompatible components (Figure 11c) [84]. The resulting HAS–Croc particle demonstrated pH-responsive photoacoustic imaging and photothermal therapy, because Croc has interchangeable forms dependent on pH. As an anionic basic form in high pH, Croc exhibited a strong absorption at 680 nm; however, in the zwitterionic acidic form in low pH, strong absorption was observed at 810 nm. Thus, they could monitor relatively large tumors in detail and ablate them effectively. Figure 11d,e depict the π-conjugated dyes based on a phenazine–cyanine structure, where the phenazine moieties donate electrons, while the indole moieties withdraw electrons. Owing to the push–pull effect, the dyes have a narrow band gap and absorb NIR light, which facilitates photoacoustic imaging-guided photodynamic therapy. The dyes aggregated with human serum albumin (HAS), which enabled the formation of nanoparticles that have appropriate sizes to be easily accumulated in tumors in mice by enhanced permeability and retention (EPR) and treat cancer tissues effectively [85].

**Figure 12:**
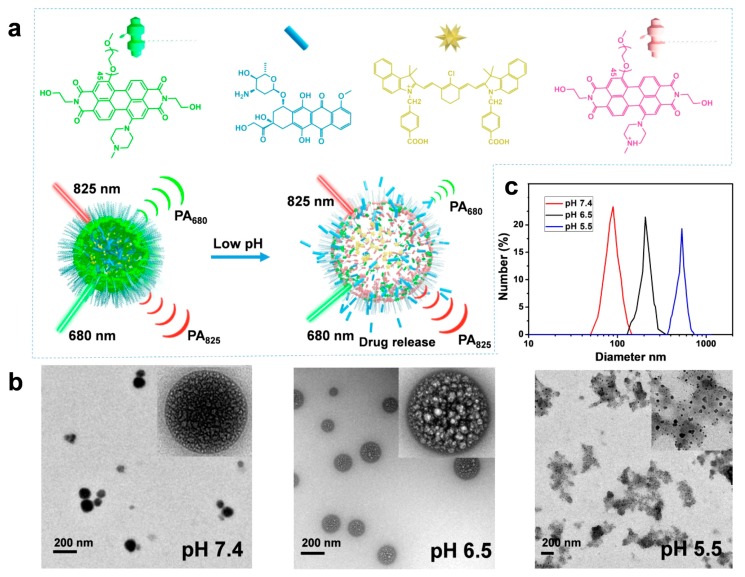
Characterization of the theragnostic platform (THPDINs). (**a**) Schematic illustration of the sensing and drug-releasing mechanism of THPDIN. The THPDIN is self-assembled with a pH-sensitive protonated PDI (HPDI, green), a pH-inert IR825 (gold), and an anticancer drug of DOX (blue). At low pH, the HPDI will be protonated (pink), inducing a loosened nanostructure that could trigger the release of the encapsulated DOX accompanied by PA signals vanishing at 680 nm. Meanwhile, the chemical structure of IR825 and its characteristic PA signal at 825 nm retain the same. Therefore, the DOX release process could be monitored by ratiometric PA imaging at PA825/PA680. (**b**) TEM images and (**c**) DLS data indicate diameters of the THPDINs in buffer solutions with different pH values. Reprinted with permission from [86]. Copyright, Ivyspring International Publisher (2019).

Recently, Chen et al. demonstrated theragnostic platforms (THPDINs) that are comprised of a pH-sensitive perylene diimide derivative (HPDI). The molecule further self-assembled with IR light-absorbing dye (IR825) and anti-cancer doxorubicin (DOX) to form particles. Upon the change in pH, the particles could be dissembled under mild acidic conditions while the absorption spectrum of HPDI changed, which accompanied the triggered release of DOX and also enabled ratiometric photoacoustic imaging due to the deliberate inclusion of IR825 (Figure 12) [86]. The authors found that the theragnostic system was in vitro or in vivo effective to U87MG glioma cell line and U87MG tumor model.

**Figure 13:**
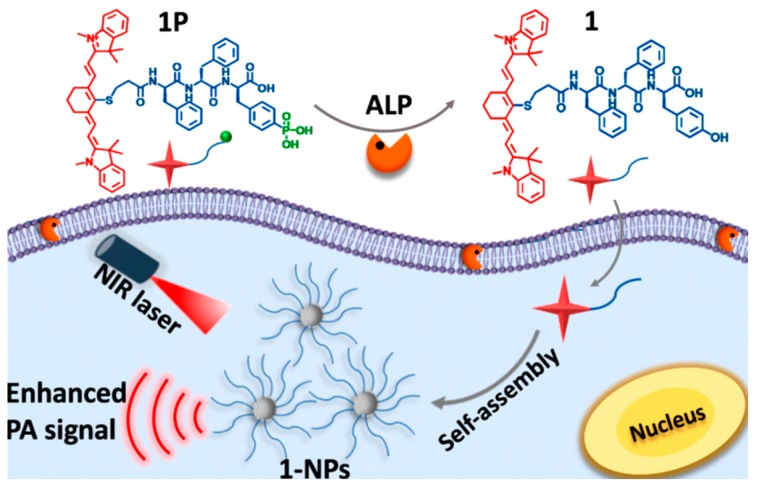
Schematic illustration of alkaline phosphatase (ALP)-triggered self-assembly of NIR nanoparticles from **1P** (1) for the enhanced photoacoustic imaging of tumors. Reprinted with permission from [87]. Copyright, American Chemical Society (2018).

Liang et al. recently demonstrated the rational design of a functional PA probe that is responsive to alkaline phosphatase (ALP). The probe **1P** has an NIR-absorbing moiety (IR775) and a phosphate group. When exposed to the enzyme, dephosphorylation sensitively occurred, which triggered the rapid self-assembly of resultant molecule **1** due to the hydrophobic effect. Then, the assembled particle was able to demonstrate an enhanced PA signal. Given that certain tumors, such as SK-OV-3 and ATDC5, secrete at low levels, this approach would provide precise diagnoses to discern the types of cancers (Figure 13) [87].

#### 4.2.3. Other Semiconducting Materials

**Figure 14:**
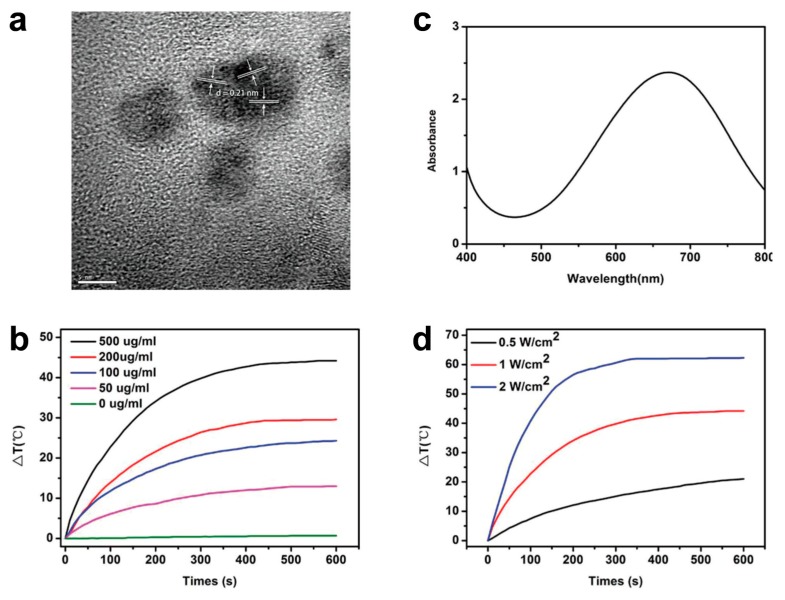
(**a**) High-resolution transmission electron microscopy (HR-TEM) image of supra-carbon nanodots (CNDs). (**b**) Absorption spectra of supra-CNDs in aqueous solution. (**c**) Temperature evolution of various concentrations of supra-CND solutions under 655 nm laser irradiation at a power density of 1 W cm^−2^. (**d**) Temperature evolution of supra-CND solutions (0.5 mg mL^−1^) at various power densities. Reprinted with permission from [88]. Copyright, John Wiley and Sons (2019).

Carbon materials can be used for photoacoustic imaging as well as photothermal conversion. As depicted in Figure 14 [88], Qu et al. investigated supra-carbon nanodots (supra-CNDs) that are formed by the self-assembly of surface charge-confined CNDs by electrostatic force or hydrogen bonding. The materials exhibited well-developed absorption in the NIR region, and could be accumulated in tumor tissues in mice when measured by in vivo PA imaging after intravenous injection. Furthermore, the following photothermal therapy efficiently inhibited tumor growth, which has paved the way for biomedical PA application of carbon-based materials.

### 4.3. NIR-II Imaging Contrast Agents

#### 4.3.1. Semiconducting Polymers

**Figure 15:**
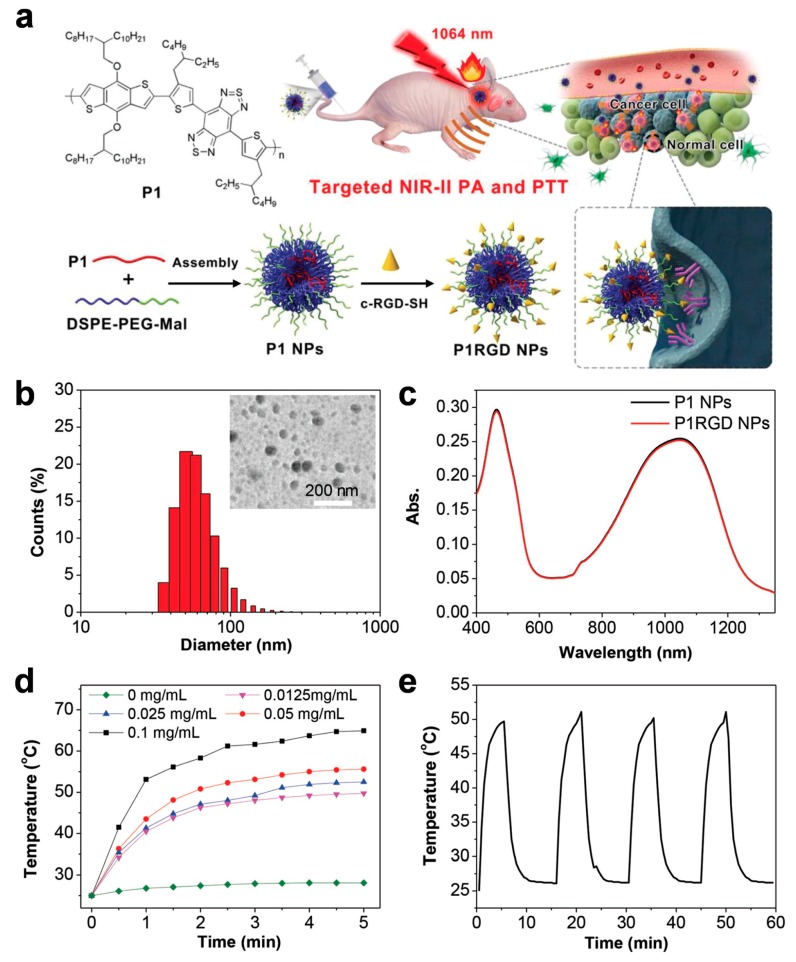
(**a**) Illustration of nanoparticle fabrication and *in vivo* brain tumor photothermal therapy and photoacoustic imaging. (**b**) Dynamic light scattering (DLS) data and transmission electron microscopy (TEM) image of P1RGD NPs. (**c**) UV–Vis spectra of P1 NPs and P1RGD NPs, respectively. (**d**) Photothermal heating effects of P1 NPs at different concentrations under a 1064-nm laser (1 W cm^−2^). (**e**) Cyclic photothermal heating and cooling of P1 NPs (0.01 mg mL^−1^). Reprinted with permission from [92]. Copyright, John Wiley and Sons (2018).

Very recently, photoacoustic imaging using NIR light in the second window (NIR-II, 1000–1700 nm) has attracted considerable attention, as NIR-II has distinct advantages, such as deeper penetration depth, higher sensitivity, and better resolution in comparison with NIR-I imaging, enabling in vivo deep-tissue imaging [89,90]. In the past, the shortage of contrast agents restricted the use of NIR-II imaging; however, now NIR-II fluorophore materials have been developed [91]. Figure 15 depicts the preparation of nanoparticles based on a low-band-gap polymer. The polymer was encapsulated with a biocompatible shell polymer, DSPE-PEG2000-MAL, which consists of an aliphatic stearyl chain, a PEG chain, and maleimide to form nanoparticles (Figure 15b), and the resulting particles exhibited a strong absorption in the NIR-II region as intended (Figure 15c). After facile nanoprecipitation, the resulting particles were then tethered with oligopeptides (c-RGD-SH) by Michael addition reaction as a targeting moiety to α_V_β_3_ integrin receptors, which are expressed in endothelial cells of the brain tumor angiogenic vasculature, as well as on glioblastoma cells. The polymeric agent enabled not only precise PA imaging but also spatiotemporal photothermal therapy, as depicted in Figure 15d,e. Therefore, the use of a 1064-nm laser resulted in more efficient penetration of the scalp and skull, and provided more effective treatment of brain tumors than the common 808-nm laser [92].

**Figure 16:**
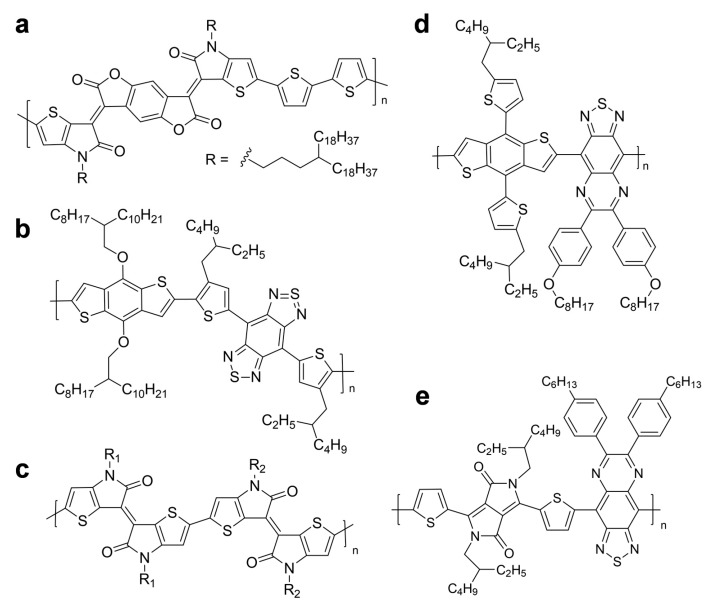
Chemical structures of other semiconducting polymers for NIR-II photoacoustic imaging.

Chemical structures of other notable semiconducting polymers are illustrated in Figure 16. In general, thiophene-based polymers contain the donor–acceptor-type structures that facilitate the hybridization of energy levels because of the push–pull effect, and demonstrate the reduced band gap that results in absorption in the NIR-II window. Figure 16a depicts the semiconducting polymer that consists of a thiophene donor and a benzodifurandione-based acceptor. The polymer was further processed to nanoparticles via nanoprecipitation, providing NIR-II PA imaging and photothermal therapy as well under 1064 nm irradiation [93]. The combination of benzodithiophene (BDT) and benzobisthiadiazole (BBT) produced a semiconducting polymer that exhibited an extremely strong donor–acceptor strength, as depicted in Figure 16b [94]. The resultant polymer was used as the core material of nanoparticles, and provided highly efficient PA imaging for orthotopic brain tumors. A thienoisoindigo (TII)-based semiconducting polymer was introduced by Mei et al. (Figure 16c) [95]. The nanoparticles from the polymer demonstrated a wide NIR-II absorption range from 1000 to 1350 nm and a deep penetration depth of over 5 cm when measured on the chicken-breast tissue, which minimized the background signal interference. Bian et al. investigated the use of thiadiazoloquinoxaline moiety [96]. The unit demonstrated strong electron-withdrawing properties and yielded a low-band-gap polymer when polymerized with a benzothiadiazole donor, as illustrated in Figure 16d. The polymer enabled NIR-II PA imaging and tracking of stem cells with an enhanced signal-to-noise ratio compared to NIR-I imaging. Copolymerization of diketopyrrolopyrrole and thiadiazoloquinoxaline resulted in a broadband absorption ranging from NIR-I to NIR-II regions, as demonstrated by Pu et al. (Figure 16e) [97]. The semiconducting polymer provided a feasible, direct comparison of NIR-I or NIR-II PA imaging and a scientific foundation regarding the advantages of NIR-II imaging, such as enhanced resolution of imaging and deep-tissue imaging, while increasing the laser power using 1064-nm irradiation.

**Figure 17:**
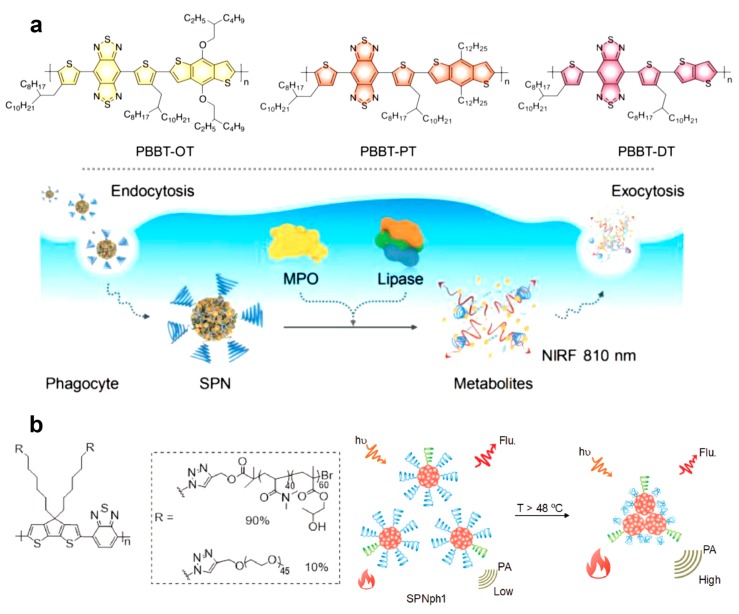
(**a**) (top) Chemical structures of semiconducting polymers, PBBT-OT, PBBT-PT, and PBBT-DT for NIR-II photoacoustic imaging, and (bottom) depiction of in vitro biodegradation of NIR-II SPNs in cells. (**b**) Chemical structure of thermoreponsive, semiconducting polymers and schematic illustration of heat-amplified PA signals of the SPNph1. Reprinted with permission from [98,99]. Copyright, John Wiley and Sons (2019).

Interestingly, Pramanik and Pu et al. demonstrated metabolizable SPNs using semiconducting polymers that enable PAI in the NIR-II window (Figure 17a). The π-conjugated polymers contain benzobisthiadiazole (BBT) that not only provides a narrow-band-gap structure with electron-donating units but is also susceptible to oxidation that brings about biodegradability. Therefore, the semiconducting polymers not only generated PA signals in response to NIR-II light, but also degraded in the presence of myeloperoxidase and lipase that are abundant in phagocytes. The SPNs were obtained via nanoprecipitation and were transformed to ultra-small, non-toxic metabolites that are further easily removed from the living mice through both renal and hepatobiliary excretions [98]. Another type of functional SPNs that exhibit heat-amplified PA signals was also demonstrated as shown in Figure 17b [99]. A semiconducting polymer was synthesized from thiophene and benzothiaziazole units, and it was further functionalized with poly(*N*,*N*-dimethylacrylamide)-*r*-(hydroxypropyl acrylate) (PDMA-r-HPA) through a graft-on approach. The resulting brushed polymers formed SPNs via self-assembly (SPNph1) and showed lower critical solution temperature (LCST) behavior due to the random copolymer tethers. While undergoing aggregation by phase transition of the polymer grafts, the large SPNs displayed enhanced PA signals that not only imparts a thermo-sensitive response but also increases the signal-to-noise ratio for high-contrast imaging.

#### 4.3.2. Semiconducting Small Molecules

**Figure 18:**
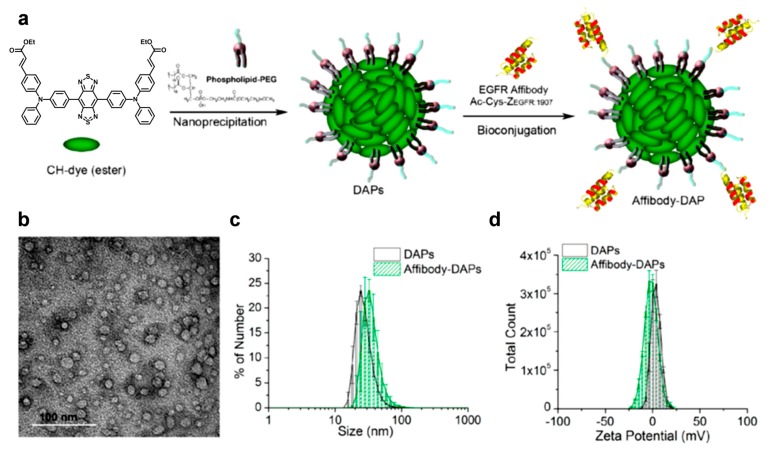
(**a**) Schematic illustration of preparation of affibody−DAPs. The DAPs were prepared through nanoprecipitation of CH1000. The CH1000 molecules are represented as light green ovals. The phospholipid (DSPE-PEG-5000) has two hydrophobic tails and one hydrophilic PEG chain, and is illustrated as a purple ball with two dark gray tails and one light gray head. EGFR affibodies (Ac-Cys-ZEGFR:1907, three α-helices) were immobilized on the surface of DAPs via a bifunctional cross-linker. (**b**) Representative TEM image of negatively stained DAPs. Scale bar = 100 nm. (**c**) Hydrodynamic sizes of DAPs (black line and column) and affibody−DAPs (green line and column). (**d**) Zeta potentials of DAPs and affibody−DAPs. Reprinted with permission from [100]. Copyright, American Chemical Society (2017).

Although most NIR-II contrast agents are based on semiconducting polymers because of their feasibility for long π conjugation, small molecules that have a strong donor–acceptor structure can also be used as NIR-II imaging agents. For example, CH1000 dye that contains a donor−π−acceptor−π−donor structure exhibited efficient PA imaging, as demonstrated by Cheng et al. [100]. The chromophore molecule (CH-dye) was synthesized using triphenylamine and benzobisthiadiazole, and encapsulated using PEG modified with phospholipid. The nanoparticles were further tethered with the antiepidermal growth factor receptor (EGFR)-affibody to target EGFR-positive cancer, and provided PA imaging and fluorescent imaging as well, leading to specific, dual-modal contrast imaging (Figure 18). 

**Figure 19:**
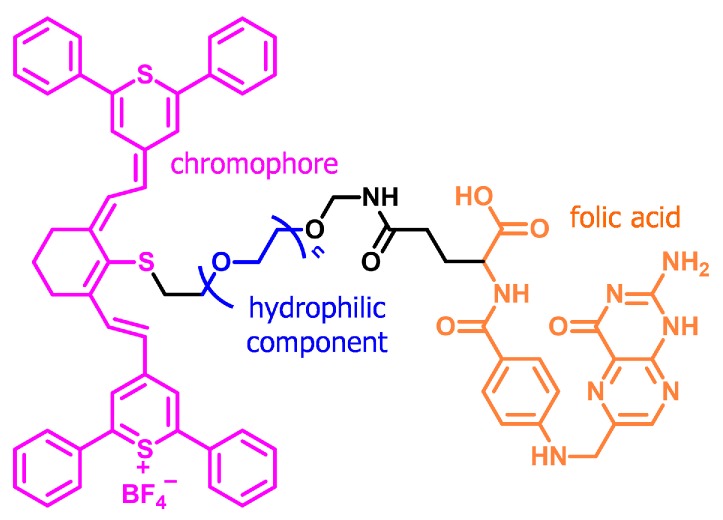
Chemical structure of a multifunctional small molecule that enables target-specific, high-resolution imaging.

More sophisticatedly, Xie et al. demonstrated a multi-modal contrast agent after the judicious chemical modification of IR-1061 dye [101]. As depicted in Figure 19, the agent not only exhibited NIR-II imaging properties due to the commercial NIR dye (pink), but also was biocompatible due to the PEG moiety (blue) and was functionalized with a cancer-targeting folic acid moiety (orange). Therefore, the designed probe enabled high-resolution imaging for the specific diagnosis of cancer.

**Figure 20:**
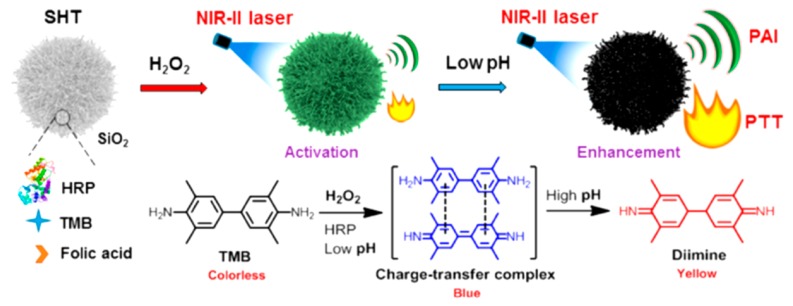
Description of formation of the tumor microenvironment-activated nanotheranostics (SHT), the activation in response to hydrogen peroxide, and acid enhancement for tumor-specific NIR-II photonanotheranostics. Reprinted with permission from [102]. Copyright, American Chemical Society (2019).

A turn-on-type PA agent notably enhanced the specificity and sensitivity of the PA signal. Figure 20 shows a stimuli-responsive, biocompatible, nanotheranostic agent that provides both photoacoustic tomography and photothermal therapy in the NIR-II window [102]. The functional agent consists of horseradish peroxidase (HRP) as an enzyme and 3,3′,5,5′-tetramethylbenzidine (TMB) as a substrate, both of which were encapsulated in a mesoporous silica container that was further tethered with folates as a tumor-targeting moiety. Thus, the catalase HRP promoted the formation of reactive radical species from H_2_O_2_ that oxidized TMB to form a charge transfer complex (CTC) that exhibited strong absorption in the NIR-II window. Therefore, the CTC substantiated the capabilities of NIR-II PAI and photothermal therapy. Furthermore, owing to the nature of CTC, the agent can be activated by external stimuli and also be pH-sensitive, thereby showing an enhanced, functional imaging performance that paves the way for the development of a “turn-on” theragnostic contrast agent.

#### 4.3.3. Other Semiconducting Materials

**Figure 21:**
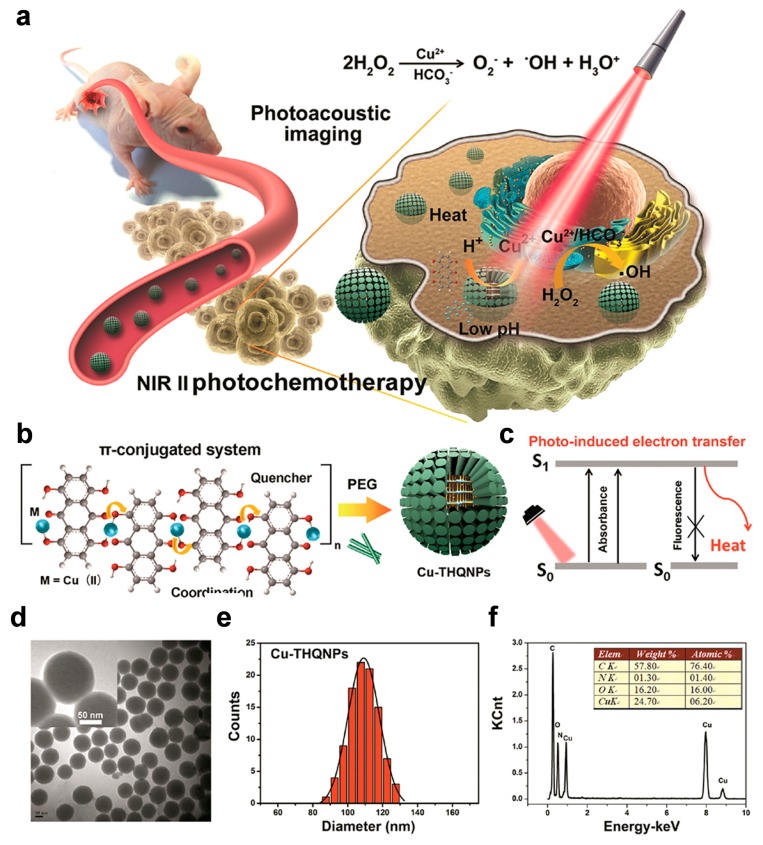
(**a**) Schematic illustration of the behavior of Cu(II)−THQNPs upon 1064-nm laser irradiation in vivo. (**b**) Synthesis of Cu(II)−THQNPs by a one-step method. (**c**) Mechanism of Cu(II)−THQNPs transforming photoenergy to heat. (**d**) Transmission electron microscopy (TEM) image of Cu(II)−THQNPs; the inset image is the enlarged picture of Cu(II)−THQNPs. (**e**) Size distribution histogram based on TEM images of Cu(II)−THQNPs. (**f**) Energy-dispersive X-ray spectroscopy (EDS) analysis of Cu(II)−THQNPs. Reprinted with permission from [107]. Copyright, American Chemical Society (2018).

Not only carbon materials, as discussed above for NIR-I imaging, but other inorganic components have been used in PA imaging applications, such as Ag_2_S nanoparticles, silicon oxide nanoparticles, and co-doped nanocrystals [103,104,105,106]. Notably, Liu et al. developed new organic–inorganic hybrid nanoparticles based on Cu(II) ions and tetrahydroxyanthraquinone (THQ) ligands (Figure 21). The copper complex nanoparticles (Cu(II)−THQNPs) absorbed the NIR light in the second window due to surface plasmon resonance, and after encapsulation with PEG, the resulting nanoparticles became biocompatible, enabled PA imaging, and also generated reactive oxygen species (ROS) from hydrogen peroxide while undergoing a Fenton-like reaction. Thus, the material played a role as a precise theragnostic agent for PA imaging-guided photochemotherapy using NIR-II light, and caused the complete prevention of a cancerous growth for 14 days without demonstrating cytotoxicity [107].

## 5. Concluding Remarks

Based on NIR light sources, PAI systems of various scales have been developed. In the case of a microscopic system for providing high-resolution images, a depth image of 11 mm or more can be realized while maintaining ultrasonic resolution by using a laser in the NIR region. For whole-body small animal imaging and clinical application, PAI systems can be applied in the examination of diseases of organs, such as breast cancer, using NIR and special ring-shaped transducers or ultrasound-based systems. PAI imaging using NIR light is expected to be applicable in a variety of basic preclinical studies, clinical diagnostics, and disease monitoring, while maintaining depth enhancement and resolution quality.

In addition to modality, the development of diverse contrast agents is of significant importance for NIR imaging applications. In general, the materials have a core–shell structure, enhance the contrast of images, and further demonstrate sophisticatedly designed functions if necessary. Polymers have played a crucial role in the construction of core–shell-type agents: semiconducting polymeric materials form the photoactive core part, which is required to absorb light in the NIR region, and biocompatible polymers encapsulate the core and render biocompatibility under aqueous conditions. In particular, many narrow-band-gap polymers that have an alternating donor–acceptor π–conjugated structure exhibit broad absorbance in the NIR region, and thus are extensively used in photoacoustic NIR imaging. In addition, small molecules with strong donors or acceptors, or inorganic materials that have broad absorption due to their characteristic electronic properties, can be used in PA imaging when irradiated by an NIR light, thereby overcoming the shortage of materials. Further inclusion of other functionality imparts the agent materials with, for example, multi-modal imaging, targeting, and chemotherapy. 

Many agent materials have been developed and widely used; however, the incorporation of other components or well-designed chemical reactions can improve the performance of PA imaging or pave the way for sought-after applications. For example, lanthanide ions can display characteristic optical or catalytic properties while being incorporated in the agents [108,109,110,111,112,113]. Additionally, self-propagating reactions, such as self-assembly or triggered head-to-tail depolymerization [114,115,116,117,118], can readily turn on–off or even amplify the PA signal. Furthermore, addition of the PA properties to various network materials, such as porous materials or hydrogels [119,120,121,122,123,124,125,126], can provide a non-destructible in situ monitoring system or facile, selective manipulation of physical properties of the networks in response to NIR.

## Figures and Tables

**Table 1:** Representative photoacoustic imaging (PAI) systems used in near-infrared (NIR) regions.

PAI System Type	Wavelength (nm)	Imaging Depth	Spatial Resolution	Detector Type (Center Frequency)	Application	Ref.
OR-PAM	800, 1064 (NIR-I, II)	>300 µm	9.4 µm	Single unfocused TR (30, 35 MHz)	Mouse retina	[51] [52]
	1046 (NIR-II)	>3.2 mm	2.9 µm	Single focused TR (50 MHz)	Mouse brain & ear	[53]
	1064 (NIR-II)	>700 µm	15 µm	Single focused TR (40 MHz)	Melanoma cell	[19]
AR-PAM	778 (NIR-I)	>30 mm	560 µm	Single focused TR (5 MHz)	Rat spleen	[54]
	850 and 1064 (NIR-I and II)	>10.3 mm	590 µm	Single focused TR (5 MHz)	Mouse whole body	[55]
	1064 (NIR-II)	>11 mm	130 µm	Single focused TR (30 MHz)	Rat lymph nodes & bladder	[56]
PACT	730, 760, 800, 850, 900 (NIR-I)	>30 mm	200 µm	256-Spherical array TR (4 MHz)	Mouse whole body	[57]
	776, 796, 820 (NIR-I)	>19 mm	250 µm	512-Ring array TR (5 MHz)	Mouse whole body	[58] [59]
	1064 (NIR-II)	>40 mm	255 µm	512-Ring array TR (2.25 MHz)	Human breast	[60]
Clinical USI/PAI	670, 700, 800 (NIR-I)	>30 mm	300 µm	128-Linear array TR (8.5 MHz)	SLN detection	[61]
	1064 (NIR-II)	>50 mm	—	128-Linear array TR (5 MHz)	Human arm	[62]
